# Sedimentary organic matter from a cored Early Triassic succession, Georgetown (Idaho, USA)

**DOI:** 10.1186/s13358-020-00205-9

**Published:** 2020-04-20

**Authors:** Elke Schneebeli-Hermann, Borhan Bagherpour, Torsten Vennemann, Marc Leu, Hugo Bucher

**Affiliations:** 1 Paleontological Institute and Museum, University of Zurich, Karl Schmid-Str. 4, 8006 Zurich, Switzerland; 2 Department of Earth Sciences, Faculty of Sciences, Shiraz University, Shiraz, Iran; 3 Institute of Earth Surface Dynamics, University of Lausanne, Géopolis, 1015 Lausanne, Switzerland

**Keywords:** Palynology, Particulate organic matter, Stable carbon isotopes

## Abstract

The plant fossil record from Lower Triassic sedimentary successions of the Western USA is extremely meager. In this study, samples from a drill core taken near Georgetown, Idaho, were analyzed for their palynological content as well as their stable carbon isotope composition. The concentration of palynomorphs is generally low. The lowermost part of the drilled succession represents Dinwoody/Woodside Formation and contains spore and pollen assemblages with Permian and Early Triassic affinity. Representatives of lycophytes (*Densoisporites* spp., *Lundbladisporites* spp.) were found in the overlying *Meekoceras* Limestone, in agreement with middle Smithian assemblages elsewhere. Ammonoids and conodonts are extremely rare, but confirm a middle Smithian age. Bulk organic and carbonate carbon isotope composition provide a stratigraphic framework. Carbonate carbon isotope compositions are compatible with the Smithian–Spathian global trend, with a middle Smithian shift towards lower δ^13^C values followed by a late Smithian shift towards higher values. Bulk organic carbon isotope compositions have been influenced by changes in the constitution of organic matter. A comparison with other paired carbon isotope datasets from the same basin is difficult due to lithostratigraphic inconsistencies (Hot Springs, ID) or biochemical mediated disturbance of isotope signals (Mineral Mountains, UT).

## Introduction

In the western United States, plant fossils from sediments straddling the Permian–Triassic boundary are scarce (McKee 1954), thus reconstructing the vegetation history during this period is impossible for this region. The scarcity of late Permian–Early Triassic plant macro- and microfossils contrasts with the rich plant fossil records from the Late Triassic Chinle Formation in the southern part of this area (New Mexico, Arizona) (Ash 1972; Litwin 1985; Litwin et al. 1991; Lindström et al. 2016).

**Fig. 1:**
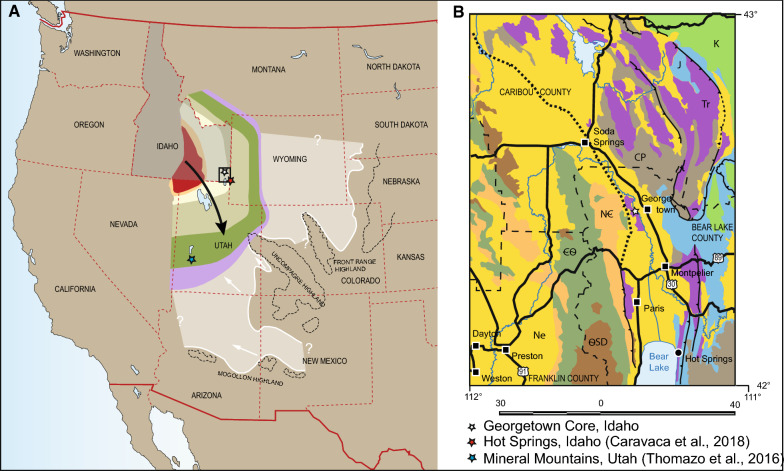
**a** Map of the Western USA (based on Google maps). The Sonoma basin is indicated after Caravaca et al. (2018), direction of the Smithian transgression after Vennin et al. (2015) and Brayard et al. (2013). Extent of the Moenkopie and Chugwater groups (pale area) as well as sediment transport directions after Stewart et al. (1972). **b** Geological map of the Bear Lake area based on Lewis et al. (2012) (Geological map of Idaho 1:750,000)

Approaching the Permian–Triassic boundary from the Palaeozoic, the youngest Palaeozoic plant macrofossils are known from the mid-Permian Blaine Formation in Texas (DiMichele et al. 2004). From Roadian–Wordian deposits of the Park City and Phosphoria formations in NE Utah a number of acritarchs have been described (Jacobson et al. 1982). Younger Permian fossiliferous deposits are not known from the Western United States (Collinson et al. 1976; Wardlaw et al. 1995). Besides the rich plant fossil record of the Late Triassic Chinle Formation, the oldest Mesozoic plant fossils have been described from the Lower–Middle Triassic Moenkopi Group. This formation stretches from SE Utah, New Mexico, Arizona and interfingers with the Thaynes Group towards the NW (Idaho) and the Chugwater Group to the N (Montana) (Kummel 1954, 1957; Stewart et al. 1972; Lucas et al. 2007) (Fig. 1). Since the early twentieth century poorly preserved plant remains, mostly impressions and a few petrified stem fossils, were known from the upper part of the Moenkopi Group (late Spathian–Anisian) (McKee 1954; Morales 1987) including sphenophytes and plant remains resembling the Palaeozoic conifer genus *Walchia* spp. (Gregory 1917; McKee 1954). Ash and Morales (1993) described Anisian plant assemblages from the Holbrook Member of the Moenkopi Group of Arizona. These belong to the oldest known Mesozoic megaflora in North America. The flora is composed of fungi, pith casts of *Neocalamites* sp., petrified fern trunks, and conifer wood resembling *Araucarioxylon arizonicum* (Ash and Morales 1993). From the late Early Triassic (?) Wupatki Member only sphenophyte stems are reported (Morales 1987). Benz (1980; in Morales 1987) was the first to document palynomorphs from late Spathian to Anisian coprolites of the Moenkopi Group in Arizona. The spores and pollen grains were poorly preserved and were assignable to Gnetales, Cordaitales, seed ferns, sphenophytes and ferns (Benz 1980 in Morales 1987). However, their identification was questioned (Litwin and Ash in Morales 1987).

During the Early Triassic the climate was suggested to have been globally influenced by the Pangean megamonsoon (Parrish 1993; Miller and Baranyi 2019). The Sonoma basin was situated on the western Pangean margin close to the equator. Continent interiors were arid to semi-arid, but not necessarily stable throughout the Early Triassic, various studies present evidence for changes in temperature and precipitation (Preto et al. 2010). The formation of red beds during the Early Triassic indicates at least seasonal precipitation (van Houten 1973; Parrish 1993). During the Triassic, the strength of the monsoonal systems may have enabled circulation patterns that are drawing moisture from the equatorial Panthalassa onto the western coast of Pangea (Parrish 1993). This provided conditions for red bed formation, and rich plant communities during the Late Triassic.

Despite the dearth of plant fossils, the lithologies of the western United States are well known for their Early Triassic marine fauna including ammonoids, conodonts, bivalves, and gastropods (Kummel 1943, 1954; McKee 1954; Müller 1956; Ware et al. 2011; Brayard et al. 2013). More recently, both fish and ichthyosaurs have also been described (Romano et al. 2012, 2017; Scheyer et al. 2014) and exceptionally diverse Early Triassic marine fauna, known as the Paris biota (Brayard et al. 2017), is found approximately 20 km South of Georgetown.

This study documents palynomorphs partly from the Dinwoody/Woodside Formation and from the lower part of the Thaynes Group recovered from samples of a drill core from a scientific borehole near Georgetown in the Bear Lake district. With respect to the scarcity of land plant fossils so far recovered in this region, these findings are illustrated and discussed. Paired carbon isotope compositions including particulate organic matter data are discussed.

### Geological setting

During the Early Triassic the Western Interior was characterized by a wide basin bordered by the Permian–Triassic Sonoma orogeny to the West (Blakey and Ranney 2018) (Fig. 1). In this shallow basin marine sediments (limestones and shales) of the Lower Triassic Thaynes Group were deposited in the western central part reaching their southeastern most extent during the Smithian transgression (Vennin et al. 2015; Caravaca et al. 2018). These marine deposits interfinger with the marginal marine to nonmarine sediments of the Moenkopi Group and Chugwater Group to the East (Fig. 1) deposited on a vast arid plain approximately at sea level with predominant deltaic and coastal plain red bed deposition (Blakey and Ranney 2018). The Thaynes Group (sensu Lucas et al. 2007) crops out in the area around Georgetown (Fig. 1). In the Bear Lake area, the Smithian–Spathian succession has been subdivided into seven units (Thaynes Formation sensu Kummel 1943, 1954) the lowermost three are included in the present study: The basal part consists of the *Meekoceras* Limestone, followed by the lower shale unit, and the *Tirolites* Limestone above. Later the Thaynes Formation has been elevated to Group rank, a marine succession correlated with the terrestrial Early to Middle Triassic Moenkopi Group (Lucas et al. 2007). In the Bear Lake area the calcareous siltstones and limestones of the Griesbachian–Dienerian Dinwoody Formation intercalated by red to maroon calcareous siltstones of the Woodside Formation (Kummel 1954) underlie the *Meekoceras* Limestone.

## Materials and methods

### Particulate organic matter and palynology

The studied succession was recovered from a drilled borehole core just above the western bank of the Bear River (coordinates: N 42° 28′ 41.0″/W 111° 24′ 50.1″), near Georgetown, Idaho. The drilling was realized in August 2016 and reached a total core depth of 151.9 m.

Fifty-four samples were selected from the Georgetown core. They were cleaned, crushed and weighed (15 g on average) and subsequently treated with concentrated hydrochloric and hydrofluoric acid as described by Traverse (2007). The residues were sieved over a 11-µm mesh screen. For palynofacies analysis organic matter particles were counted on unoxidized slides to a minimum of 250 counts. Particle counts included the categories of translucent and opaque wood (charcoal), but also degraded woody particles (pseudoamorphous particles partly with angular shape and/or remaining internal structure). Cuticles, inertinite (used for particles describable as “worn and transported oxidized or carbonized wood” Tyson 1995, p 351), membranes, spores and bisaccate pollen grains were also counted (Tyson 1995; Batten 1996). Aquatic particles include acritarchs, *Leiospheridia* and foraminiferal test linings. Usually amorphous organic matter is also included in the aquatic group of particles; however, the origin of these particles in the Georgetown core remains enigmatic.

For analysis of the spore and pollen content most samples needed further treatment. A split of the sample was treated with concentrated nitric acid, applied only to samples that have a high content in amorphous or pseudoamorphous organic matter. For these, a stepped process of oxidation (concentrated nitric acid) and ultrasonic vibration was necessary in order to increase palynomorph concentration. After oxidation the residues were sieved again over a 11-µm mesh screen.

Slides are stored in the repository of the Palaeontological Institute and Museum, University of Zurich, and are found under PIMUZ A/VI 138, PIMUZ A/VI 139, PIMUZ A/VI 140.

### Carbonate carbon and bulk organic matter carbon isotope compositions

Fifty-six samples were selected for δ^13^C_carb_ and δ^18^O_carb_ measurements on bulk micrite. Samples were carefully cleaned, cut, and finely ground. Diagenetic calcite veins, cracks, stylolites, and large skeletal particles were avoided. The C- and O-isotope composition of the carbonates were measured at the University of Lausanne (Faculty of Geosciences and Environment) with a GasBench II linked to a DeltaPlus XL mass spectrometer (ThermoFisher Scientific) according to a method adapted after Spötl and Vennemann (2003). Carbonates were reacted at 70 °C with 100% orthophosphoric acid and the extracted CO_2_ calibrated against a number of in-house Carrara Marble (CM) replicates for acid fractionation and normalization of the δ^13^C and δ^18^O values that are expressed at the permil scale. The CM standard was, in turn, calibrated against δ^13^C and δ^18^O values of NBS-19 (+ 1.95 and − 2.20 ‰, relative to VPDB). The average reproducibility of about 8 CMs analyzed in each run of 40 samples is better than 0.06‰ for δ^13^C and 0.08‰ for δ^18^O values.

Fifty-five samples were selected for bulk organic carbon isotope composition (δ^13^C_org_); all pulverized using a ceramic mortar. About 5 g of powdered samples were dissolved in 6 M hydrochloric acid to remove all carbonates. After centrifuging, residues were rinsed several times with deionized water and centrifuged until neutrality was reached. The residues were dried overnight at 45 °C and δ^13^C_org_ values of the homogenized residues were measured using a Carlo Erba 1500 elemental analyser connected to a ThermoFisher Delta V Plus mass spectrometer at the University of Lausanne. The samples were individually wrapped in tin foil cups and sequentially allowed to react with an injected quantity of oxygen while continuously flushed with He carrier gas. The sample was oxidized in the reactor at about 1050 °C using cobalt(II) oxide as the catalyst. Excess oxygen in the He-stream was adsorbed in a reactor column filled with metallic Cu held at 500 °C. The CO_2_ produced was passed over a magnesium perchlorate (Mg[ClO_4_]_2_) trap to remove H_2_O, and a gas chromatograph to separate the N_2_ from the CO_2_, before the CO_2_ is carried by the He-stream into the mass spectrometer for isotopic analysis. The reproducibility of several in-house standards used is better than 0.1‰ and they are calibrated against USGS-24 graphite (− 16.0‰ VPDB) and NBS-22 oil (− 29.6‰ VPDB).

### Conodonts

Thirteen conodont samples, each a ca. 0.5 kg sample of carbonated rock, have been selected from the core. Conodont elements were extracted using the standard buffered acetic acid technique (~ 10%, Jeppsson et al. 1999). Subsequently, a density separation with sodium polytungstate was applied in order to separate the conodont fraction (Jeppsson and Anehus 1999).

## Core description and comparison with regional lithology

**Fig. 2:**
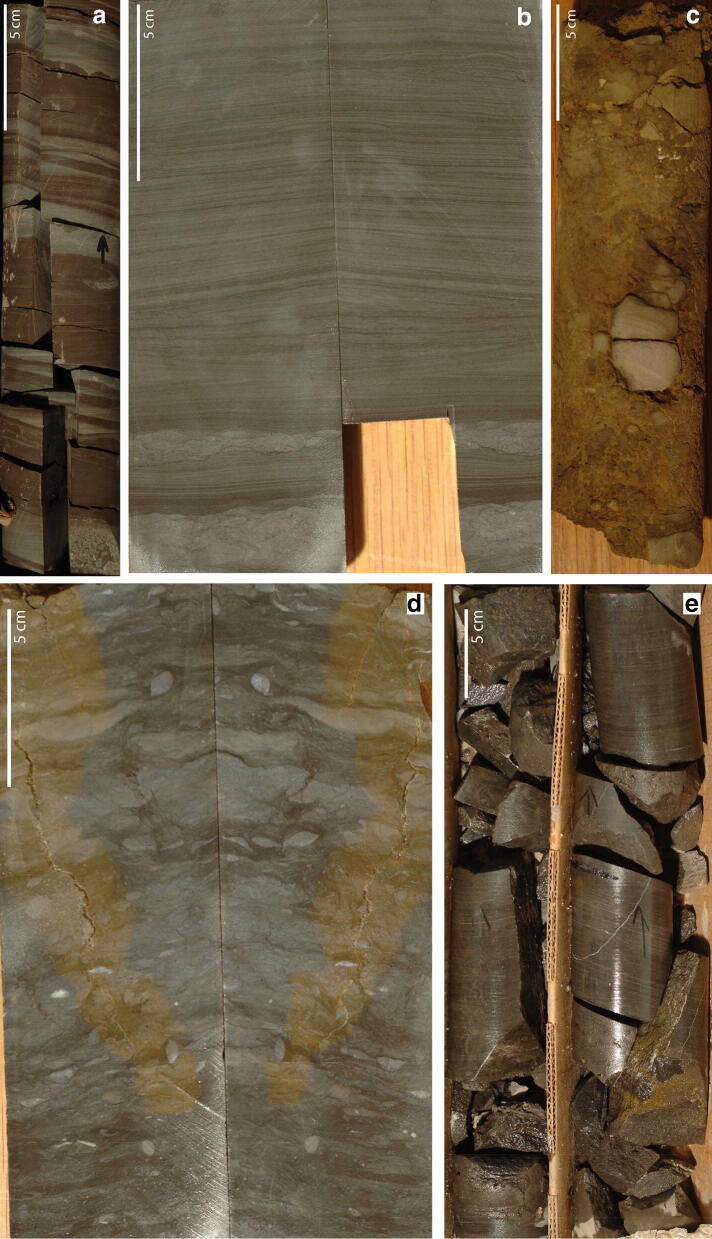
Core pictures: **a** unit I, **b** unit II, **c** karstified interval unit V, **d** Meekoceras limestone facies unit III, **e** dark shaly limestone unit IV

The lithological sequence of the core has been subdivided into five lithological intervals and are described top down (Fig. 2):

Unit V: the uppermost interval ranges from the surface to ca. 70 m core depth. The lithology of the entire unit consists of collapse breccia, characterized by heterogeneous and angular limestone clasts, yellow-to-light grey in color. The internal sedimentary structures vary from laminated to slightly bioturbated to homogeneous. The clasts are embedded in a yellow matrix (karstic loam). From 35.7 to 40.7 m and from 47.4 to 49.4 m the lithology consist of light grey-to-greenish laminated limestone. However, it is difficult to judge from the core whether these intervals are in place or just very large clasts. Exceptional is also the interval between 57.9 and 61.0 m, there the lithology of a collapse breccia is continued, but the color of the matrix changes from dark grey at the top to light grey at the base of this interval. Below collapse breccia with yellow matrix comparable to the uppermost interval prevails again down to 70.1 m.

Unit IV ranges from 70.1 to 102.1 m and consists of dark grey shaly limestone (argillaceous limestone), which is laminated. Styloliths and dissolution surfaces are present throughout but decreasing downwards. These dark grey facies is interrupted in an interval from 86.9 to 91.4 m by the occurrence of a medium grey laminated limestone.

Unit III ranges from 102.1 m down to 140.5 m. The lithology is composed of light-to-medium grey limestone in which laminated intervals alternate with nodular bioclast-rich limestone intervals of few centimeter thickness. Middle Smithian ammonoids (*Juvenites* sp.) occur at 104.2 m and around 108 m (indeterminate).

Unit II expands from 140.5 to 149.6 m. The grey limestone is marked by laminated intervals alternating with homogenous grey fine-grained bioturbated limestone intervals. Several centimeter thick lenses fully packed with bivalves (bivalve debris) occur regularly as well as styloliths and macroscopic pyrite crystals. Between 142 and 143 m the lithology shows a yellow color and is brecciated (karstified). In two horizons, ca. 25 and 50 cm above the boundary to the lower unit, mudclasts intercalated into the laminated intervals of grey limestone indicate reworking.

Unit I comprises the interval from 149.6 m to the base of the core (151.9 m). The lithology consists of shaly limestone with an alternation in color (grey and red-brown). Generally, the lithology is laminated to cross-bedded, ripples are observed. This pattern is regularly interrupted by bioturbated intervals (containing *Lingula* sp.) with flowcasts at the base and by a few-cm-thick coquinoid layers at 146.8 m and 151.3.

A comparison with previously described successions in the area of the Bear Lake district suggests that lithological unit I represents an interval in which grey calcareous siltstone and grey limestone containing *Lingula* spp. of the Dinwoody Formation and the maroon to red shaly siltstones of the Woodside Formation are intercalating (Kummel 1954). Kummel (1954, 1957) described the boundary between the Dinwoody and the Woodside formations as arcuate belt stretching from SW Montana, along the Idaho–Wyoming unfossiliferous border into NE Utah, with the Woodside Formation to the East and a belt to the west in which the Woodside formation interfingers with the Dinwoody Formation. The Woodside Formation is unfossiliferous, whereas the Dinwoody Formation is regarded Griesbachian to Dienerian in age (Clark and Carr 1984). The Dinwoody and Woodside formations are also noted to occur below the Thaynes Group in the well-studied succession at Hot Springs (ID) (Caravaca et al. 2018; Kummel 1954) ca. 45 km South of Georgetown.

Unit II marks a transition from the more siliciclastic Woodside/Dinwoody formations to the limestone dominated lower units of the Thaynes Group. Unit III is marked by the typical facies of the *Meekoceras* beds, however, these beds are usually between 15 and maximum 30 m thick in Southeastern Idaho (Kummel 1954). At Hot Springs these beds show a thickness of ca. 15 m (Caravaca et al. 2018; Kummel 1954). In Georgetown, however, the facies resembling the *Meekoceras* limestone is nearly 40 m thick indicating that the boundary between the Dinwoody/Woodside formations and the Thaynes Group might be diachronous. This is supported by variable thicknesses of this facies of up to 45 m at Fort Hall (ID, West of the Georgetown area) and in the Swift Creek (WY, East of Georgetown) succession (Kummel 1957). The uppermost part of lithological unit III contains *Juvenites* sp. an ammonoid genus restricted to the *Meekoceras* limestone in SW Idaho (Kummel 1954) and indicative of middle Smithian age (Brühwiler et al. 2012). The argillaceous limestone of unit IV corresponds to the Lower Shale of Kummel (1943). 3.6 km west of Georgetown, Kummel (1954) described the occurrence of ammonoids belonging to the *Anasibirites* Zone (late Smithian). With about 30 m thickness unit IV (Lower Shale, Kummel 1943) is less thick compared to Hot Springs, where this interval accounts for 70 m of sedimentary succession (Kummel 1943). According to Kummel (1943, 1954), the thickness of this interval is variable to some extent reaching 88 m in the Spring Canyon (WY) succession (Kummel 1954). A more recent study measured 150 m of Lower Shale in the Hot Springs succession including a 130-m-thick covered interval contrasting with significantly less expanded intervals in older lithological descriptions (Kummel 1943) and illustrations of the succession (Jenks et al. 2013).

The karstified limestone of Unit V most probably represents the Spathian *Tirolites* Limestone (Kummel 1943, 1954). However, ammonoids have not been found in this unit in the Georgetown core.

## Results

### Particulate organic matter

**Fig. 3:**
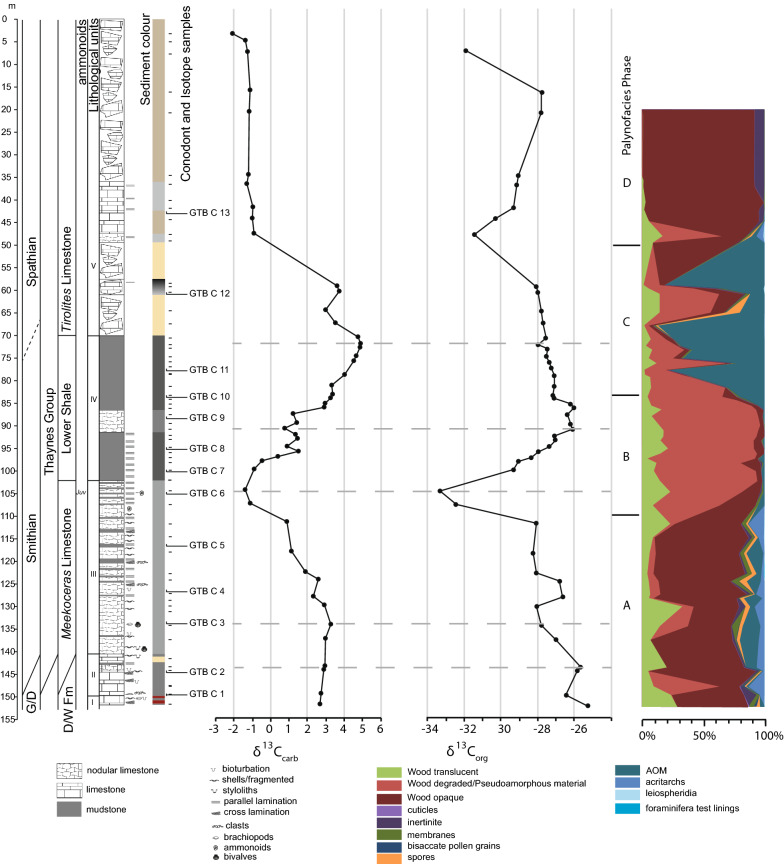
Lithological column, stable organic and carbonate carbon isotope, and particulate organic matter

**Fig. 4:**
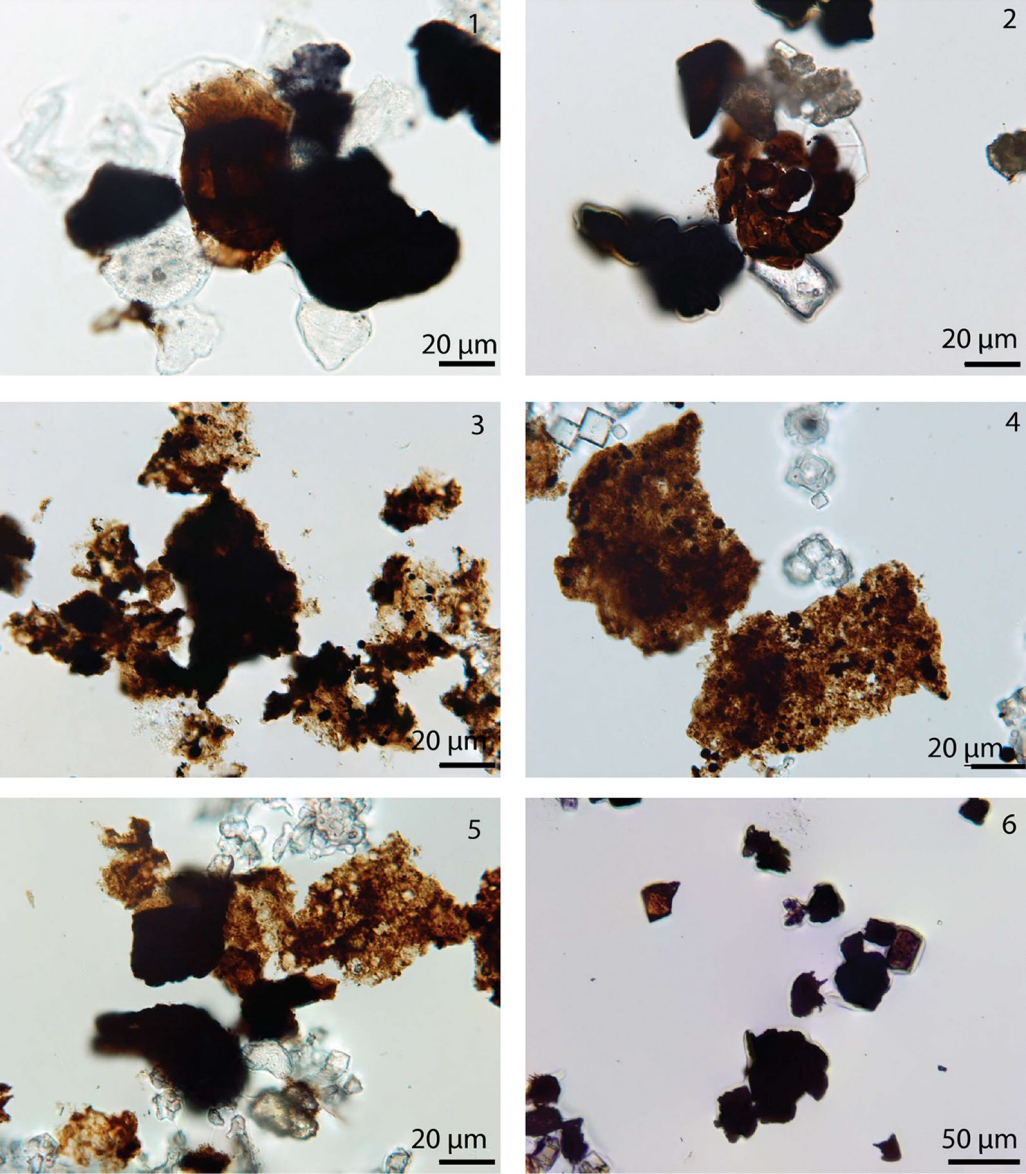
Different particulate organic matter compositions in the Georgetown core succession: **1** Kerogen GTB 001 K a 12.2/123.3; **2** Kerogen GTB 004 K a 10.5/126.3; **3** Kerogen GTB 020 K a 20.7/128.3; **4** Kerogen GTB 036 K a 6.7/135.1; **5** Kerogen GTB 048 K a 9.4/141.5; **6** Kerogen GTB 052 K a 19.0/129.5

The particulate organic matter (POM) shows four phases with distinctive composition (Fig. 3). Phase A defines the base of the core to 111.45 m (sample GTB 015) and thus encompass lithological units I, II and most of unit III. The POM assemblages are marked by the dominance of opaque and translucent wood particles. In two samples, degraded wood is present in higher amounts. Additionally, bisaccate pollen grains are observed especially in the lower part of phase A and spores in the middle and upper part. Acritarchs and amorphous organic matter each contribute several percent to the POM assemblage throughout phase A (Fig. 4 1, 2).

Assemblages of phase B are documented for samples between 108 and 84 m core depth. POM assemblages of this phase contain a continued stable contribution of translucent wood. In contrast to the previously described phase, phase B is dominated by pseudoamorphous organic matter. Residual structures in some particles suggest that these particles represent degraded wood. Fluorescence is not observed in these particles. Opaque wood and amorphous organic matter occur in minor amounts. Acritarchs occur extremely sporadically (Fig. 4 3, 4).

In phase C, which encompasses the interval between 84 and 50 m of the core, amorphous organic matter is dominant with the exception of samples GTB 043 and GTB 044. These show a similar composition as the assemblages of phase B. POM phase C includes the upper part of lithological unit IV and the lower part of lithological unit V.

POM phase D coincides with the uppermost 50 m of the core and therefore corresponds to lithological unit V. Except for one sample (GTB 048, 47.68 m), POM assemblages are dominated by opaque wood particles and inertinite. Sample GTB 048 contains additionally degraded wood, a small amount of spores, membranes, amorphous organic matter, and acritarchs (Fig. 4 5, 6).

### Palynology

**Fig. 5:**
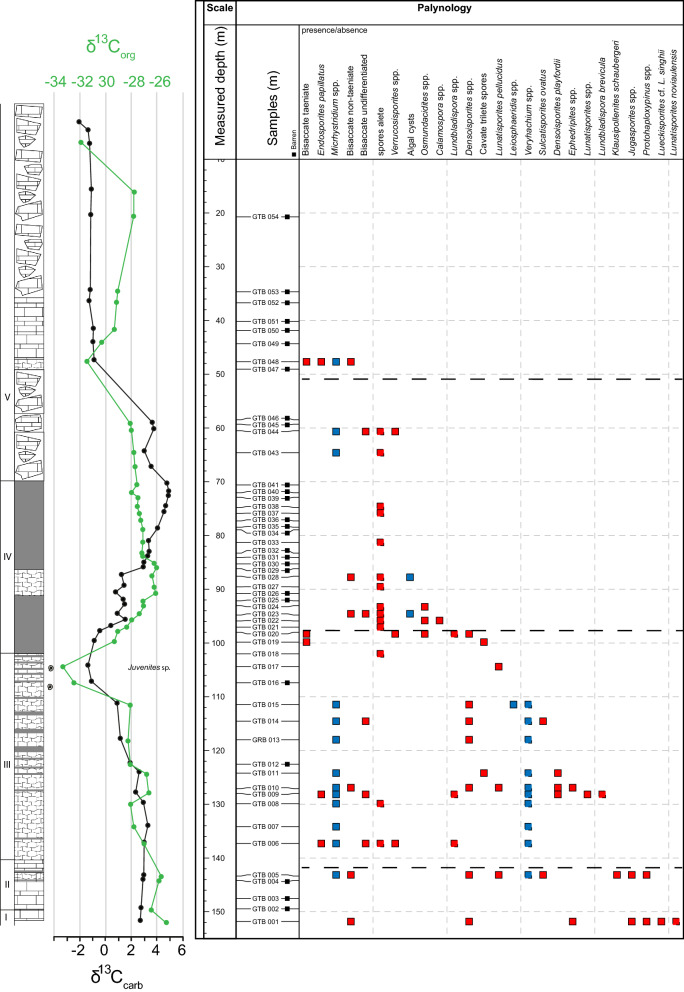
Presence/absence data of palynomorphs ordered according to their stratigraphic last occurrence. Each square represents only one or maximum two occurrences of the respective palynomorph

Generally, palynomorphs are preserved only in very low quantities. From 54 samples prepared for palynological analysis 28 turned out to be barren and in the remaining 26 samples palynomorphs are very rare. The preservation varies throughout the succession. The palynomorphs are reasonably well preserved in the lower part, and poorly preserved in the rest of the succession—in most cases they are not determinable. Analysis of relative abundances of spore and pollen taxa was thus impossible; Fig. 5 displays therefore just occurrence data, each dot representing maximum of 2 occurrences. Spore and pollen color is very dark indicating thermal maturation (5/6 on the thermal alteration scale Batten 1982). Acritarchs usually show the same dark brown color, but some lighter ones have been observed as well. In GTB 006 and GTB 009, acritarchs preserved remnant fluorescence.

**Fig. 6:**
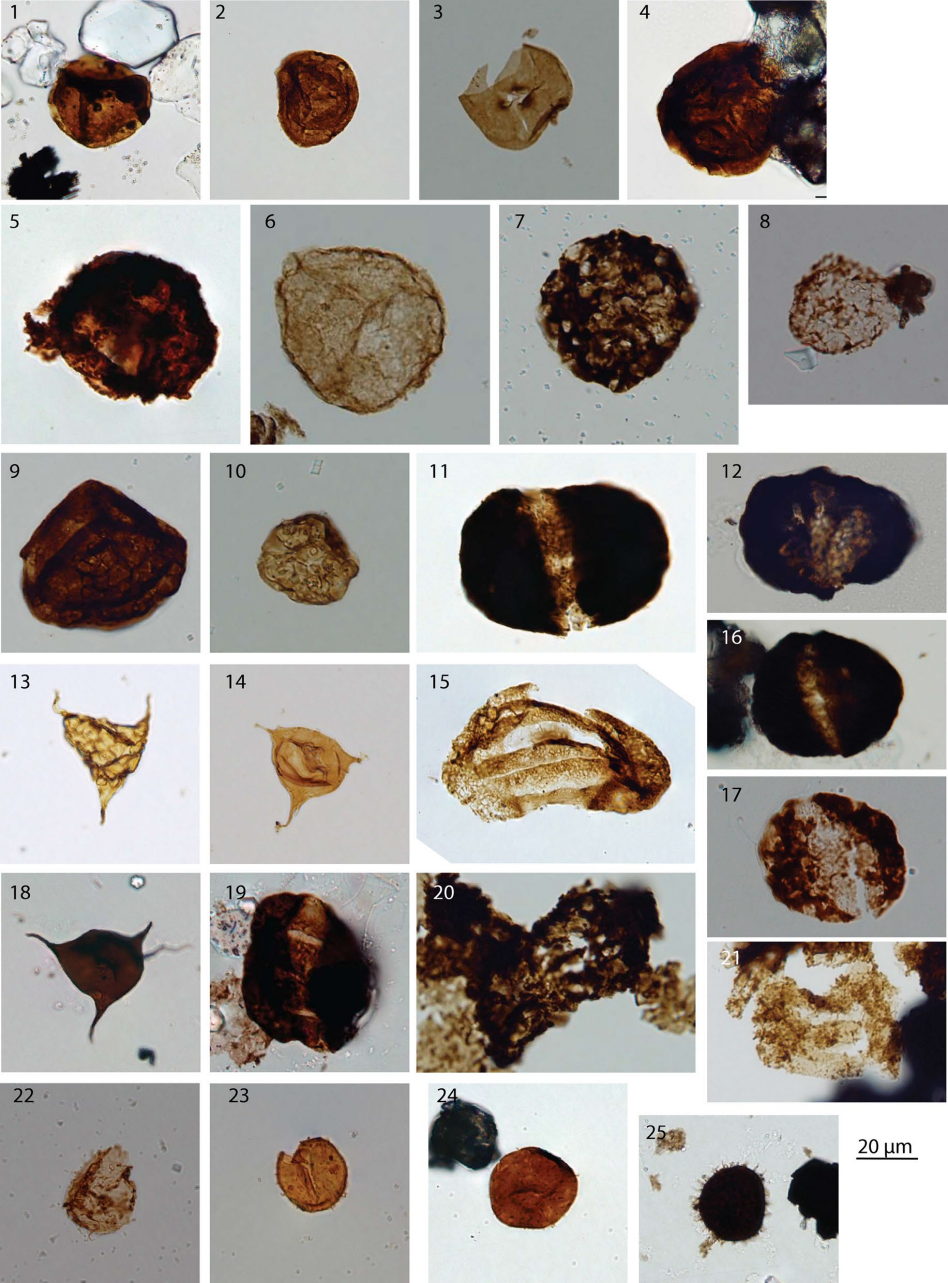
Palynomorphs recovered from the Georgetown core. **1** *Densoisporites nejburgii* GTB 014 K a 10.1/127.5; **2** *Densoisporites playfordii* GTB 009 ox a 5.5/145.0; **3** *Endosporites papillatus* GTB 009 ox a 16.2/117.5; **4** *Lundbladispora* cf. *L. brevicula* GTB 009 ox a 10.5/141.9; **5** *Verrucosisporites* sp. GTB 044 ox a 5.4/120.0; **6** relict cavate spore? GTB 015 ox a 10.8/118.8; **7** corroded palynomorph GTB 043 ox a 20.2/128.3; **8** poorly preserved spore GTB 038 ox a 10.9/127.5; **9** *Densoisporites* sp. GTB 013 ox a 16.2/123.3; **10** trilete spore GTB 006 ox a 14.9/141.0; **11** *Lueckisporites* cf. *L. singhii* GTB 001 K a 8.2/118.2; **12** non-taeniate bisaccate pollen grain GTB 001 K a 13.8/127.9; **13** *Veryhachium* sp. GTB 015 ox a 11.4/145.5; **14** *Veryhachium* sp. GTB 009 ox a 9.9/143.5; **15** *Lunatisporites* sp. GTB 009 ox a 15.6/123.7; **16** *Protohaploxypinus* sp. GTB 001 K a 16.9/135.0; **17** *Sulcatisporites ovatus* GTB 048 ox a 5.9/142.8; **18** *Veryhachium* sp. GTB 005 K a 18.2/147.0; **19** *Lunatisporites pellucidus* GTB 005 K a 7.8/127.2; **20** taeniate bisaccate pollen grain GTB 020 ox a 11.6/111.4; **21** *Lunatisporites pellucidus*? GTB 017 ox a 12.3/134.7; **22** *Micrhystridium* sp. GTB 044 ox a 11.9/140.2; **23** *Micrhystridium* sp. GTB 009 ox a 20.5/148; **24** *Micrhystridium* sp. GTB 009 ox a 18.5/136.9; **25** *Micrhystridium* sp. GTB 005 K a 14.9/135.2

In the lowermost lithological units I and II palynomorphs are very dark brown but little corroded. Thus, several taxa could be identified. *Densoisporites* sp. occurs together with *Ephedripites* sp. *Jugasporites* sp., *Lueckisporites* cf. *singhii*, *Lunatisporites noviaulensis*, and *Protohaploxypinus* sp. in the lowermost sample (GTB 001) (Fig. 6). The next productive sample above (GTB 005) covers partly the palynological content of GTB 001 and additionally contains *Klausipollenites schaubergeri*, *Lunatisporites pellucidus*, *Sulcatisporites ovatus* and acritarchs (*Micrhystridium* spp. and *Veryhachium* sp.). Sporomorphs and acritarchs are non-fluorescent.

Palynomorphs recovered from lithological unit III include *Endosporites papillatus*, *Lundbladispora* spp. (*L. brevicula*), *Lunatisporites* sp. *Densoisporites playfordii*, *Densoisporites nejburgii*, undeterminable spores and pollen grains, as well as consistent occurrence of *Micrhystridium* sp. and *Veryhachium* sp. Spore color is lighter compared to palynomorphs of unit I and II. Acritarchs especially the lighter ones are still fluorescent. In sample GTB 009 there are fluorescent and non-fluorescent acritarchs present.

Sporomorph preservation is extremely poor in lithological unit IV. The sporomorphs recovered best assignable to *Osmundacidites/Verrucosisporites*.

The only productive sample from lithological unit V is GTB 048 it contains *Densoisporites* sp., *Endosporites papillatus*, taeniate and non-taeniate bisaccate pollen grains and *Micrhystridium* spp.

### Carbon isotope compositions

The *carbonate* δ^13^C values start at the base of the core with values of around 2.7‰ and increase slightly by a 0.5‰ across lithological units I, II and the basal part of unit III towards a turning point at 137.17 m. From there values show a gentle trend to lower values up to the cored depth of 111.46 m, followed by a decrease to the minimum value of − 1.4‰ at 104.4 m in the top part of unit III. In the basal part of unit IV carbonate carbon isotope values increase again to values around 1‰ at 95.9 m and remain fairly constant until they increase again from 87.57 m upward. The most positive values of 4.9‰ are reached in the topmost part of unit IV at a core depth of 72.05 m. The maximum is followed first by a 1‰ decrease, followed by a slight increase in δ^13^C values again in the interval between 72.05 and 59.3 m (unit V). δ^13^C values of the topmost 50 m of the core are characterized by rather homogeneous values of around − 1‰ (Fig. 3).

*Bulk organic* δ^13^C values decrease continuously from − 25.3‰ at the base of the core to − 28.1‰ at 111.45 m. In the upper part of lithological unit III, values decrease to a minimum of − 33.3‰ at 104.4 m. In lithological unit IV bulk organic carbon isotope values increase to values of − 26.1‰ at 90.81 m and remain stable for ca. 5 m before they decrease by about 1‰. Values across the upper part of lithological unit IV and the lower part of unit V are marked by a slight continuous decrease from − 27‰ at 84.1 m to − 28‰ at 59.3 m. In the uppermost part of the core, bulk organic δ^13^C values decrease to − 31.5‰, but rise again to − 27.7‰ before they decrease again to − 31.9‰ in the topmost measured sample (GTB 056) (Fig. 3).

### Conodonts from the drill core in Georgetown

From the 13 investigated samples, only four samples yielded conodont elements, the remainder were all barren.

GTB C2 and GTB C3 yielded single unidentifiable ramiform elements. Sample GTB C6 consisted of several different ramiform and segminate P1 elements, including three specimens which could be identified as *Neospathodus* cf. *spitiensis* (Goel 1977), a typical cosmopolite (middle) Smithian species (Shigeta et al. 2009).

A single broken P1 element of a segminiplanate *Neogondolella*? sp. was recovered from sample GTB C 10.

## Discussion

### Particulate organic matter

The dark brown color of palynomorphs and the absence of any fluorescence indicate a mature-to-postmature hydrocarbon stage. Acritarch fluorescence in some cases in the lower part of the studied succession does not contradict a rather high thermal maturity (Hartkopf-Fröder et al. 2015; ca 170 °C).

Organic matter in the lower half of the Georgetown core is derived predominantly from the terrestrial realm. However, a contrast in preservation appears between phases A and B. Organic matter is less well preserved in phase B, which might be the result of OM remobilization and re-deposition, whereas phase A is characterized by well-preserved terrestrial OM. A different source of terrestrial OM in these two phases cannot be excluded. The change from phase A to phase B occurs in the upper part of the *Meekoceras* Limestone facies of the Thaynes Group. The meaning of the amorphous organic matter in phase C remains enigmatic. Either these particles represent even less well preserved terrestrial organic matter compared to phase B or alternatively, they are marine in origin but poorly preserved (no fluorescence despite “only” brown color). In this second option, their abundance could be compatible with the marine transgression and main flooding zone documented from the Mineral Mountains succession in Utah (Vennin et al. 2015). The uppermost part is influenced by karstification and contains residual organic matter, i.e., opaque particles less affected by degradation.

### Palynology

In lithological units I and II taxa occurring characteristically in Permian deposits such as *Lueckisporites* spp., (here: *L*. cf. *singhii*), *Klausipollenites schaubergeri,* and *Protohaploxypinus* spp. are mixed with typical Early Triassic forms such as *Densoisporites* spp. or *Lunatisporites* spp., especially *L. noviaulensis* and *L. pellucidus*. The taxonomic composition of assemblages thus shows a mixed Late Permian–Early Triassic character. Similar assemblages with mixed Permian–Triassic character are known from the basal Wordie Creek Formation at Kap Stosch (Greenland, Schneebeli-Hermann et al. 2017) or from the basal Flagstone Bench Formation (Antarctica, Lindström and McLoughlin 2007). Samples from both these localities included all above-mentioned genera and at least two of the mentioned species. The basal Wordie Creek Formation as well as the basal Flagstone Bench Formation are regarded to be Griesbachian in age (Lindström and McLoughlin 2007; Schneebeli-Hermann et al. 2017). Additional support for a correlation with other lowermost Triassic palynological assemblages comes from conodont-based Griesbachian–Dienerian age of the Dinwoody Formation (Clark and Carr 1984).

Reworking of typical Permian taxa cannot be excluded; however, there is no distinct difference in preservation between the “Permian” taxa and the “Early Triassic” taxa, and underlying Permian deposits in the area (Phosphoria and Park City formations) are so far known to contain only acritarchs (Jacobson et al. 1982). Thus, Permian sediments as possible source for those taxa are currently unknown.

Palynomorphs of lithological unit III are in agreement with Dienerian and Dienerian to Smithian assemblages elsewhere (e.g., Wordie Creek Formation, Greenland, Schneebeli-Hermann et al. 2017; Balme 1979; Rewan Formation, Bowen Basin, Foster 1982, Mianwali Formation, Salt Range, Pakistan Hermann et al. 2012). Shared taxa in these localities and unit II of the Georgetown core are *Lunatisporites* spp., *Densoisporites* spp. (*D. nejburgii, D. playfordii*), and *Lundbladispora* spp. (*L. brevicula*). Thus, the palynological assemblages were not very differentiated in a belt from 30° N to 40° S. (Bear Lake area = equator; Pakistan 30° S, Greenland 30° N, Bowen Basin ca 40° S, after Stampfli and Borel 2002). Time equivalence with Dienerian and Dienerian to Smithian palynological assemblages is supported by the occurrence of the middle Smithian ammonoid *Juvenites* and conodont *Neospathodus* cf. *spitiensis*, together with carbon isotope stratigraphy.

#### Components of the vegetation

The core was drilled in the fully marine central part of the Sonoma Basin. Spores and pollen grains have been washed into this basin from the catchment area in the West (Fig. 1), elevated areas of the Umcopahgre Highland and the Salt Anticline Region, as well as the Defiance Uplift and the Mogollon Highland. The marginal marine Moenkopi Group was deposited between these elevated areas and the central Sonoma basin (Thaynes Group) and thus even closer to the plant habitats, but the plant fossil record is scanty nonetheless. Palynomorphs have a higher preservation potential compared to macrofossils, but also they are hardly preserved in the oxygenated depositional environment indicated by red beds of the Moenkopi Group. Thus, the presence of palynomorphs in the Thaynes Group is remarkable which represent plants or plant groups which shall be illustrated here.

In lithological units I and II spores and pollen grains indicate the presence of lycophytes (*Densoisporites* spp.), Gnetales (*Ephedripites* spp.), conifers of the families Ulmanniaceae and Majonicaceae (*Jugasporites* spp., *Lueckisporites* spp.), other conifers (*Lunatisporites* spp.), and seed ferns of the order Peltaspermales (*Protohaploxypinus* spp.). Palynofacies data display a strong terrestrial signal during this time, indicating continental runoff and proximity to the shoreline (Fig. 3). In the following, lithological unit III slightly increased marine influence is indicated, but still terrestrial influence is dominant. The interval is characterized by the presence of lycophytes, mainly Pleuromeiaceae (*Densoisporites* spp., *Lundbladisporites* spp.). These plants have been interpreted as either pioneer plants or halophytes living under semi-arid condition close to water bodies (Naugolnykh 2013; Retallack 1997).

### Carbon isotope compositions

Early Triassic carbon isotope records are marked by significant fluctuations worldwide (e.g., Corsetti et al. 2005). The Smithian–Spathian transition is characterized by the occurrence of a prominent negative excursion during the middle Smithian followed by a positive excursion straddling the Smithian–Spathian boundary (e.g., Payne et al. 2004; Galfetti et al. 2007a, b; Zhang et al. 2019). However both records, organic and inorganic, are not necessarily reflecting secular carbon cycle changes, but rather diagenetic overprinting, compositional changes of locally derived organic matter or other environmental factors that may have influenced the local environment only (e.g., Popp et al. 1997; Arthur et al. 1985; Edwards and Saltzman 2016; Bagherpour et al. 2019).

**Fig. 7:**
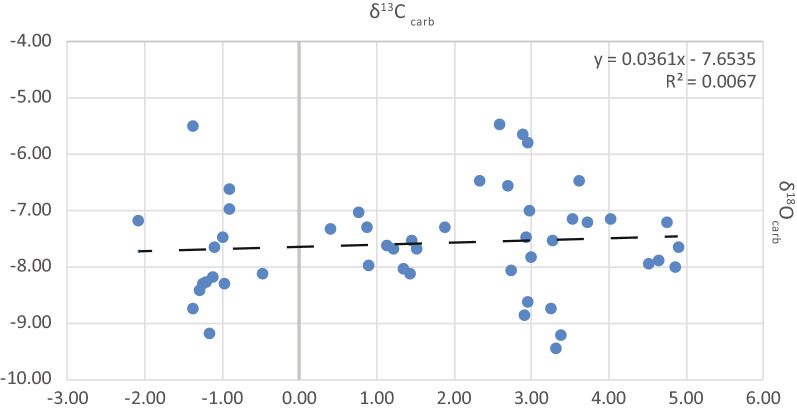
δ^13^C_carb_–δ^13^C_org_ crossplot

Bulk carbonate carbon isotopes are a reliable proxy for oceanic DIC (dissolved inorganic carbon) and hence global CO_2_ isotopic changes in open marine systems when diagenetic overprinting can be excluded (e.g., Kump et al. 1999; Weissert et al. 2008). For the Georgetown core, δ^13^C–δ^18^O crossplots (Fig. 7) show no covariance and thus would support the interpretation that the stable carbon isotope compositions were not overprinted by diagenesis. Furthermore, the isotopic composition of DIC is influenced by the bioproductivity at the surface, competing with organic matter respiration at increasing depths down to the oxygen minimum zone. In modern oceans this phenomenon is responsible for a vertical carbon isotope gradient of DIC of about 2‰ (depending on the bioproductivity/nutrient supply and oxygen content), with higher δ^13^C_DIC_ values at the surface and lower values at increasing depths towards the O_2_ minimum zone (Hilting et al. 2008). This will be discussed below in a comparison of other paired carbon isotope datasets from the same basin.

One of the most important effects on bulk organic carbon isotopes are changes in the composition of organic matter. Carbon isotope compositions of terrestrial and marine organic matter can differ by several per mil (e.g., Arthur et al. 1985; Tyson 1995; Foster et al. 1997). Thus, changes in the contribution of either marine or terrestrial organic matter sources can have a fundamental impact on the δ^13^C curve. In addition, the aquatic organic matter often changes in concert with the DIC in the same water column, as this is the principal nutrient for aquatic biosynthesis. Particulate organic matter C-isotope compositions indicate significant differences between phase A, B, and C. The first marked compositional change from phase A to B concerns the preservation of terrestrial organic matter: well preserved opaque wood vs. poorly preserved, i.e., pseudoamorphous terrestrial OM. δ^13^C of modern terrestrial OM is known to increase by up to 2‰ during early diagenetic degradation, notably because of the generally more rapid degradation of isotopically lighter proteins compared to other carbohydrates such as lignin more common in woody tissues (Tyson 1995). However, a systematic analysis of opaque and the pseudoamorphous particles encountered in the Georgetown core has not been performed. Additionally, the negative shift from A to B is opposite to what would be expected for a signal derived from organic matter preservation with remaining woody tissue (lignin) being isotopically rather heavy compared to proteins. The second significant shift in OM composition between phase B and C might have a bigger influence on bulk OM composition. Pseudoamorphous OM is replaced by amorphous OM. While a marine origin of the amorphous OM cannot be proven, an increase in its relative abundance coincides with an attenuation of the positive δ^13^C_org_ shift. Thus, the compositional change might explain the different stratigraphic positions of positive peaks in organic and carbonate carbon isotope records.

Two localities have been studied previously in the Sonoma basin, the succession from the Mineral Mountains (Utah, Thomazo et al. 2016) and one from the Bear Lake area, at Hot Springs (Idaho, Caravaca et al. 2018). The paired organic and carbonate carbon isotope records from these sites show slightly differing aspects. The succession from Mineral Mountains (Utah, Thomazo et al. 2016) deposited more proximally compared to the Georgetown area illustrate negative–positive couplet in the carbonate carbon isotope record similar to what is observed in the Georgetown cores. In contrast, the organic carbon curve parallels the negative shift noted for the carbonates, but it remains rather stable up-section in the Mineral Mountains succession. This might be explained by its more proximal facies where the upper parts of the sections are more influenced by terrestrial organic matter. For Hot Springs, the negative and positive shifts in the paired carbon isotope data from carbonates and organic matter are almost synchronous to those for Georgetown, but not of the same magnitude. The Georgetown record as the third but most distal record in this basin, displays its own peculiarities. The middle Smithian negative shift and the position of the minimum are shared in organic and inorganic carbon isotope compositions. During the positive shift while the records remains parallel in their direction of change, the two curves drift apart, with the δ^13^C_org_ values increasing more profoundly compared to the δ^13^C_carb_ values; the latter also reach their maximum value later. As previously mentioned δ^13^C_org_ values might be influenced by the compositional change in OM with marine and terrestrial OM having different values too and thus the δ^13^C_org_ positive peak precedes the one of δ^13^C_carb_.

Assuming a vertical carbon isotope DIC gradient similar to modern day oceans it could be expected that the shallower succession (Mineral Mountains) should have overall heavier C-isotope compositions compared to Hot Springs and Georgetown as shallower, coastal sections often have a higher nutrient supply from terrestrial inputs and hence a higher bioproductivity. However, the Mineral Mountains section has overall lower δ^13^C_carb_ values. Pre-excursion values of δ^13^C_carb_ range around 0‰ in Mineral Mountains and around 2‰ in Hot Springs and Georgetown. Thomazo et al. (2016) argue that the δ^13^C_carb_ from the Mineral Mountain succession does not reflect global changes in carbon isotopes composition of atmospheric CO_2_ but was influenced by authigenic carbonate formation in these shallow sedimentary settings and hence perhaps also by basin-derived DIC sources. These carbonates precipitated from fluids with a ^13^C-depleted composition because of bacterially mediated organic matter remineralization either within the catchment or within the local water column (Thomazo et al. 2016). In contrast to Mineral Mountains, the δ^13^C_carb_ and δ^13^C_org_ curves from Hot Springs mirrors those for Georgetown, with a minimum in the Meekoceras beds and a positive peak in the lowermost Spathian (Caravaca et al. 2018). However, if the lithologic log from Hot Springs is plotted at the same scale as Georgetown, the picture is rather contorted because of the 130 m covered interval in the Lower Shale. The difference with Georgetown is that the δ^13^C_carb_ and δ^13^C_org_ curves remain parallel. Thus, the organic carbon isotope data seem not to be influenced by compositional changes of the OM, as would be the case for Georgetown cores.

## Conclusions

The absence or scarcity of plant fossil from Lower Triassic deposits in the Western USA does not necessarily imply an Early Triassic desert, i.e., the absence of vegetation during this time in this region. Palynomorphs recovered from a drilled borehole core near Georgetown, Idaho straddling the uppermost Dinwoody/Woodside formations and the lower part of the Thaynes Group indicate the presence of lycophytes including Pleuromeiaceae, Gentales, Ulmanniaceae, Majonicaceae, and Peltaspermales. Even though the data is scares, spore and pollen grains prove the presence of different plant groups in Western United States, which have further implications for palaeopyhtogeographic and climatic considerations. The rather unexpected findings of preserved palynomorphs in Early Triassic deposits there, hopefully encourages palynological research not to neglect seemingly hopeless successions. Carbonate carbon isotope compositions have similar trends across the Smithian–Spathian boundary as in other worldwide distributed successions. Paired carbon isotope compositions of inorganic and organic carbon from three profiles of the same basin are interpreted to reflect a combination of differences in the influence of local variations in the catchment, as reflected by the lithostratigraphic differences. As such, more proximal settings suggest a stronger influence of terrestrially derived organic matter as well as DIC, while more distal settings are more influenced by differences in marine versus terrestrial proportions in the composition of the organic matter.

## Data Availability

Slides are stored in the repository of the Palaeontological Institute and Museum, University of Zurich. The datasets generated during the current study are available from the corresponding author on request.
